# Influence of frailty status on the incidence of intraoperative hypotensive events in elective surgery: Hypo-Frail, a single-centre retrospective cohort study

**DOI:** 10.1016/j.bja.2024.10.050

**Published:** 2025-01-24

**Authors:** Nils Daum, Laerson Hoff, Claudia Spies, Anne Pohrt, Annika Bald, Nadine Langer, Jörn Kiselev, Nils Drewniok, Maximilian Markus, Oliver Hunsicker, Rudolf Mörgeli, Björn Weiss, Dario von Wedel, Felix Balzer, Stefan J. Schaller

**Affiliations:** 1 Charité – Universitätsmedizin Berlin, Corporate Member of Freie Universität Berlin and Humboldt Universität zu Berlin, Department of Anaesthesiology and Intensive Care Medicine (CCM/CVK), Berlin, Germany; 2 Charité – Universitätsmedizin Berlin, Corporate Member of Freie Universität Berlin and Humboldt Universität zu Berlin, Institute for Biometry and Clinical Epidemiology, Berlin, Germany; 3 Fulda University of Applied Sciences, Department of Health Sciences, Fulda, Germany; 4 Charité – Universitätsmedizin Berlin, Corporate Member of Freie Universität Berlin and Humboldt Universität zu Berlin, Institute of Medical Informatics, Berlin, Germany; 5 Medical University of Vienna, Department of Anaesthesia, Intensive Care Medicine and Pain Medicine, Clinical Division of General Anaesthesia and Intensive Care Medicine, Vienna, Austria

**Keywords:** frailty, geriatric anaesthesia, haemodynamics, hypotension, risk assessment

## Abstract

**Background:**

Frailty is a predictor of morbidity and mortality in older patients. This study aimed to investigate the influence of frailty status on likelihood, rate, duration, and severity of intraoperative hypotension (IOH), which can lead to severe organ dysfunction.

**Methods:**

Surgical patients (≥70 yr old) with preoperative frailty assessment were analysed retrospectively. Frailty status was defined as robust, prefrail, or frail based on modified Fried criteria. IOH was defined as mean arterial pressure <65 mm Hg. For likelihood, rate, duration, and severity of IOH, logistic and Poisson regression were used.

**Results:**

We included 2495 patients. There was no significant difference in likelihood of IOH. An increase of 9% in rate of IOH during surgery for prefrail (incidence rate ratio [IRR] 1.09 [95% CI 1.03–1.16], *P*=0.002), and 16% increase for frail patients (IRR 1.16 [1.04–1.29], *P*=0.007) was observed. During anaesthesia induction, prefrail patients exhibited a 28% increase in IOH (IRR 1.28 [1.12–1.47], *P*<0.001). Although there were no differences in the severity of IOH if surgery or anaesthesia induction duration was taken into account, frailty status was associated with a 15% longer time-weighted duration of IOH during anaesthesia induction (IRR 1.15 [1.06–1.24], *P*=0.001). Mediator analysis revealed that frailty status accounted for >90% after considering number of measured blood pressures and surgical duration and >70% after accounting for total propofol dose.

**Conclusions:**

Prefrail and frail patients aged ≥70 yr experienced up to 16% more IOH during surgery and 28% more during anaesthesia induction compared with robust patients. Preoperative optimisation (prehabilitation) and modification of intraoperative management (e.g. invasive blood pressure management) have the potential to reduce IOH in prefrail and frail patients.

**Editor's key points**

• Frailty is a predictor of morbidity and mortality in older patients, and could impact the likelihood, rate, duration, and severity of intraoperative hypotension.
• This retrospective cohort study of 2495 robust, prefrail, and frail patients aged more than 70 yr with preoperative frailty assessment analysed for likelihood, rate, duration, and severity of intraoperative hypotension defined as mean arterial pressure <65 mm Hg.
• Although there was no difference in the likelihood, there was a 9% increase in rate of intraoperative hypotension for prefrail and 16% increase for frail patients.
• These findings require verification in prospective studies, and suggest that prehabilitation and invasive blood pressure management could reduce intraoperative hypotension in prefrail and frail patients.

The frailty syndrome represents a state of reduced physiological reserve and provides insight into how various conditions impact individual function. Frailty is a significant predictor of surgical morbidity and mortality in older patients undergoing surgery.^1^, ^2^, ^3^, ^4^ It remains unclear which pathophysiological processes are influenced by frailty syndrome and how these determine patient outcomes.

Intraoperative hypotension (IOH) is a critical risk factor during surgery, especially in older patients with comorbidities.^5^ IOH commonly occurs in noncardiac surgeries, and even brief episodes are linked to severe negative consequences such as acute kidney injury, stroke, myocardial infarction, and an overall increase in mortality.^6^, ^7^, ^8^ A particularly vulnerable phase for IOH has been identified during the induction of anaesthesia. In a retrospective study, a considerable proportion of all hypotensive events occurred before surgical intervention and could be attributed to anaesthetic management.^9^ This is mainly explained by the vasodilatory effects of drugs used during anaesthesia induction. Rooke and colleagues^10^ demonstrated that a significant portion of decrease in blood pressure is owing to decreased systemic vascular resistance.

Only one study has explored the relationship between frailty status and intraoperative haemodynamics. In a retrospective cohort study, James and colleagues^11^ investigated the correlation between frailty, intraoperative blood pressure variability, and 30-day mortality. Their findings suggested that frailty is linked to decreased blood pressure variation during surgery, and absolute changes in mean arterial pressure (MAP) played a mediating role in connecting frailty to 30-day mortality. However, it remains unclear whether frailty status not only affects the variability of MAP but also influences the rate of decreased MAP (i.e. increased incidence of IOH).

Given the lack of evidence regarding the impact of frailty status on IOH, we aimed to investigate the influence of frailty status on the occurrence, rate, duration, and severity of hypotensive events during elective surgeries and induction of anaesthesia.

## Methods

The Charité – Universitätsmedizin Berlin Ethics Committee approved the protocol on April 6, 2023 (EA1/227/16). This study was conducted following the ethical guidelines of the Declaration of Helsinki and adheres to the applicable Equator guidelines.^12^^,^^13^

### Patient population and inclusion criteria

This was a retrospective single-centre cohort study at a major tertiary academic medical centre (Charité – Universitätsmedizin Berlin) involving the analysis of electronic medical records between December 3, 2012, and August 18, 2023. Inclusion criteria comprised electively operated adult patients aged ≥70 yr with a completed preoperative frailty assessment, comprehensive blood pressure measurements, and surgeries with induction times up to 120 min and surgery times up to 10 h. Anaesthesia induction times were calculated according to local protocols at our study centre and included the period from the start of induction until all medical measures necessary for the perioperative course (e.g. placing of a central venous catheter) were performed. If patients had multiple surgeries, repeat surgeries were only included in the analysis if they involved a new hospital stay, the subsequent surgery was performed at least 3 months after the last procedure, and a completely new frailty assessment was conducted before surgery. For multiple surgeries during the same hospital stay, only the first surgery after the frailty assessment was included.

Preoperative frailty assessment has been routinely conducted in our department as a standard screening tool for all patients aged ≥70 yr undergoing elective surgery.^14^ Frailty screening was based on the Fried criteria, encompassing domains such as unintentional weight loss ≥5 kg within the previous year, reduced grip strength, exhaustion, slow walking speed, and low activity (metabolic equivalent tasks [METs] <3).^14 15^ These criteria were developed and validated within the framework of the Cardiovascular Health Study and are used in many different clinical settings.^15^, ^16^, ^17^, ^18^ Minor modifications were made to the Fried frailty assessment to adapt and improve data collection according to European standards, as described (Supplementary Table S1).^19^^,^^20^ For each category, 1 point could be assigned, with a maximum of 5 points. A score of 0 defined the robust patient group; 1–2 points indicated a prefrail status, and a score of ≥3 indicated a frail status. Exclusion criteria included surgery involving cardiopulmonary bypass. Further, patients lacking data related to BMI, ASA physical status, and METs were excluded and the complete-case approach was used.

### Measurements and data handling

Arterial blood pressure measurements could be conducted noninvasively (NIBP) using a blood pressure cuff or invasively (IBP) via arterial access. The interval for NIBP measurement could be individually adjusted and is typically set between 2 and 5 min at our centre. IBP measurement involved continuous monitoring. IBP values are transferred to the electronic data system at discontinuous intervals. Transmission occurred at least every 2 min for both intermittently and continuously measured blood pressures.

### Endpoints

Endpoints were the occurrence (any hypotensive event), rate (proportion of hypotensive measurements), duration, and severity (area under MAP <65 mm Hg) of hypotensive events during both surgery and anaesthesia induction. Previous studies have shown that blood pressure <65 mm Hg was associated with an increased risk of end-organ damage.^7^ Therefore, hypotensive events were defined as individual blood pressure measurements with a MAP <65 mm Hg.^21^^,^^22^

### Statistical analyses

Summary statistics were generated for continuous variables utilising the median (interquartile range) for non-normally distributed data. Categorical or ordinal variables are presented using frequencies (percentages). Normality was assessed using the Kolmogorov–Smirnov test. Univariate and multivariate logistic regression analyses were conducted to investigate the association between frailty status and the likelihood of the occurrence of hypotensive events during surgery and anaesthesia induction. Odds ratios were calculated to represent the impact of the frailty status compared with the robust patient group. For analysis of the relationship between frailty status and the rate, duration, and severity of hypotensive events during surgery and anaesthesia induction, univariate and multivariate Poisson regression analyses were performed, and the incidence rate ratio (IRR) was determined. The duration and severity of hypotensive events were considered both as absolute values and in relation to anaesthesia induction time or the duration of surgery. Confidence intervals were set at 95%. In multivariate analyses, the following variables were included as confounding variables: age, ASA physical status, and BMI. The Charlson Comorbidity Index (CCI), which showed collinearity with ASA physical status according to the Pearson correlation coefficient, was not included in the multivariate analysis. As sensitivity analysis, the aforementioned logistic and Poisson regression analyses were repeated considering only the first surgery per patient compared with the robust patient cohort and with coherent measurements counted as one IOH event. We also conducted analyses for different MAP values (<60 mm Hg, <55 mm Hg, and <50 mm Hg).

To investigate the direct effect of the frailty status on the rate of hypotensive events, mediator analyses were performed on variables causally linked to the frailty syndrome and the rate of hypotensive events. Analysed parameters included the number of blood pressure measurements during surgery, the duration of surgery, the total dose of propofol, fluids, norepinephrine use, and surgery departments. The total effect was normalised to 100%, and both the direct and mediator effects were calculated proportionally. The significance level was set at *P*<0.05. Python (version 3.6.15) was used for statistical analysis with the following packages: matplotlib,^23^ statsmodels,^24^ and scipy.^25^

## Results

### Study cohort

**Fig 1:**
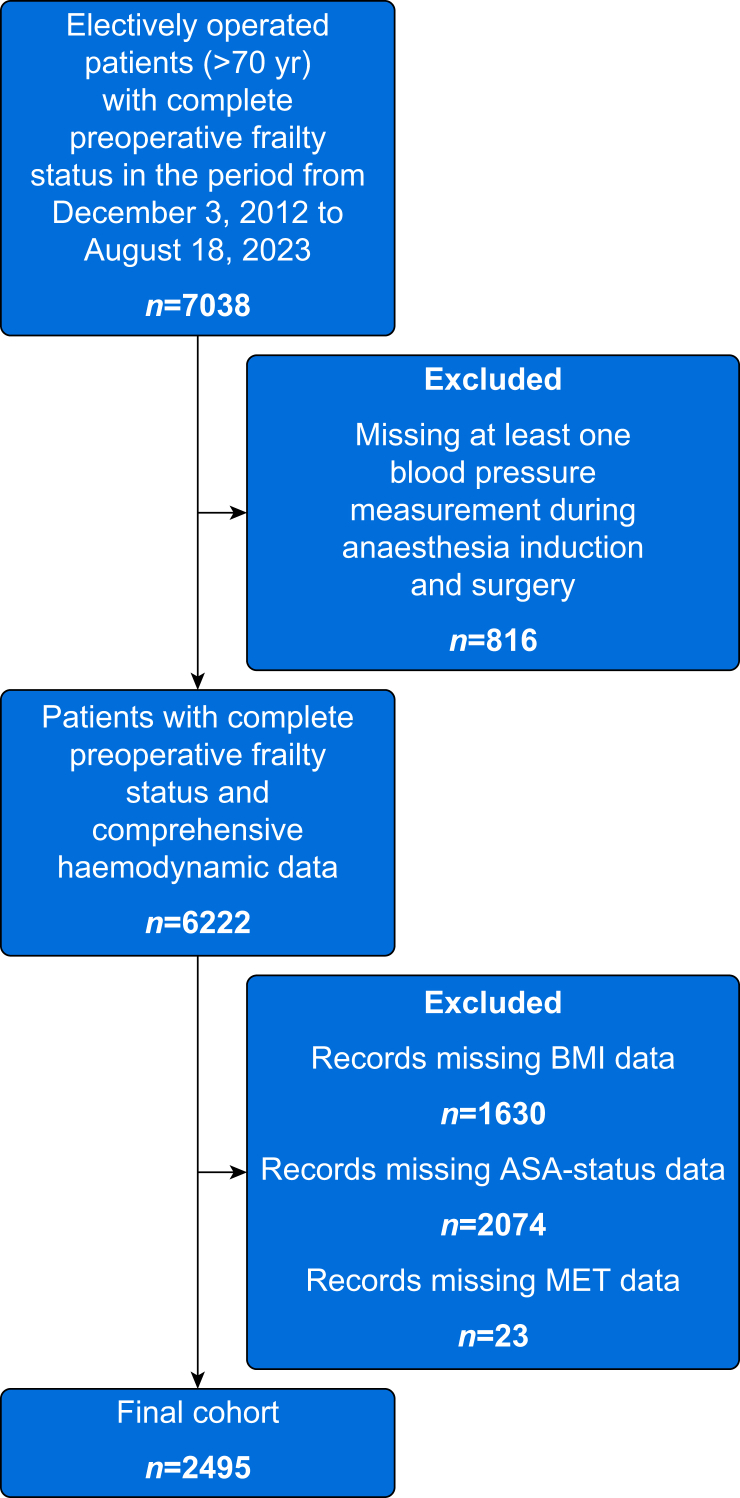
Flowchart of included patients. MET, metabolic equivalent of task.

**Figure S1:**
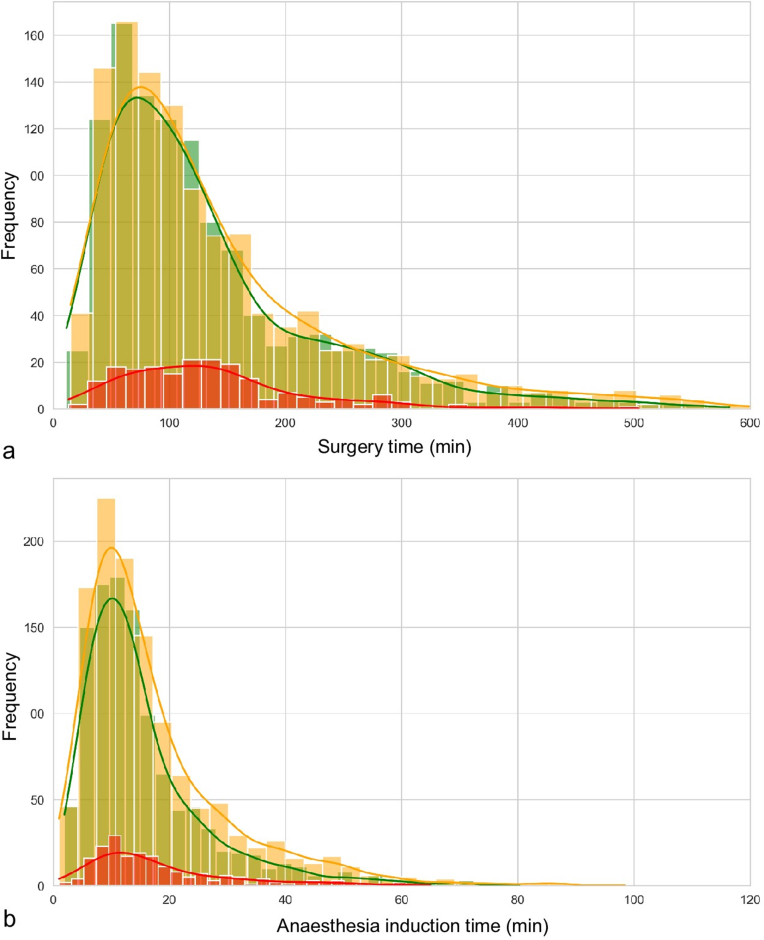
Total surgery (a) and anaesthesia induction time (b). *green* robust; *yellow* prefrail; *red* frail; *coloured lines* Kernel Density Estimation.

A total of 6222 patients (≥70 yr old) with a complete preoperative frailty assessment and comprehensive haemodynamic data (blood pressure measurements at intervals of at least every 5 min) underwent elective surgery in the study period (Fig. 1). After excluding patients according to the above-described criteria, 2495 patients with 2516 surgeries were included in the statistical analyses. Among them, 1116 (44.7%) patients were categorised as robust, 1184 (47.5%) had prefrailty status, and 195 (7.8%) had frailty status. The overall cohort age was 77 (73–81) yr, with 52% and 47% of males in the entire and frailty cohorts, respectively. The median total duration of surgery was 108 (67–168) min, with a median induction time of 13 (9–20) min. Total surgery and anaesthesia induction times are depicted in Supplementary Figure S1. Additional patient and surgical characteristics are presented in Table 1.

**Table 1:** Baseline patient, anaesthesia, and surgical characteristics. Data are presented as median (interquartile range). Categorical or ordinal variables are presented as frequencies or percentages. CCI, Charlson Comorbidity Index; MET, metabolic equivalent of task.

Category	All (*n*=2495)	Robust (*n*=1116)	Prefrail (*n*=1184)	Frail (*n*=195)
Age (yr)	77 (73–81)	76 (72–80)	78 (74–82)	79 (75–83)
Sex (% male)	51.7	56.1	48.4	46.7
BMI (kg m^−2^)	25.8 (23.0–29.0)	25.8 (23.3–28.4)	25.7 (22.7–29.3)	26.2 (22.7–30.9)
ASA physical status	2 (2–3)	2 (2–3)	2 (2–3)	3 (2–3)
MET	5 (4–6)	5 (4–6)	4 (3–5)	3 (2–4)
CCI	0 (0–0)	0 (0–0)	0 (0–0)	0 (0–1)
Surgeries (*n*)	2516	1124	1196	196
Anaesthesia induction time (min)	13 (9–20)	13 (8–19)	13 (9–22)	14 (10–23)
Surgery time (min)	108 (67–168)	106 (64–168)	108 (67–185)	125 (82–166)
Total amount of fluid (ml kg^−1^ min^−1^)	0.1 (0.0–0.3)	0.1 (0.0–0.4)	0.1 (0.0–0.3)	0.1 (0.0–0.3)
Patients receiving propofol (%)	74.8	76.8	73.1	73.5
Propofol amount (mg kg^−1^)	1.8 (1.1–2.4)	1.8 (1.1–2.5)	1.8 (1.2–2.4)	1.6 (1.0–2.4)
Patients receiving norepinephrine (%)	47.5	43.4	50.1	58.5
Norepinephrine amount (μg kg^−1^)	0.3 (0.2–0.6)	0.3 (0.1–0.5)	0.3 (0.2–0.6)	0.3 (0.2–0.7)

**Figure S2:**
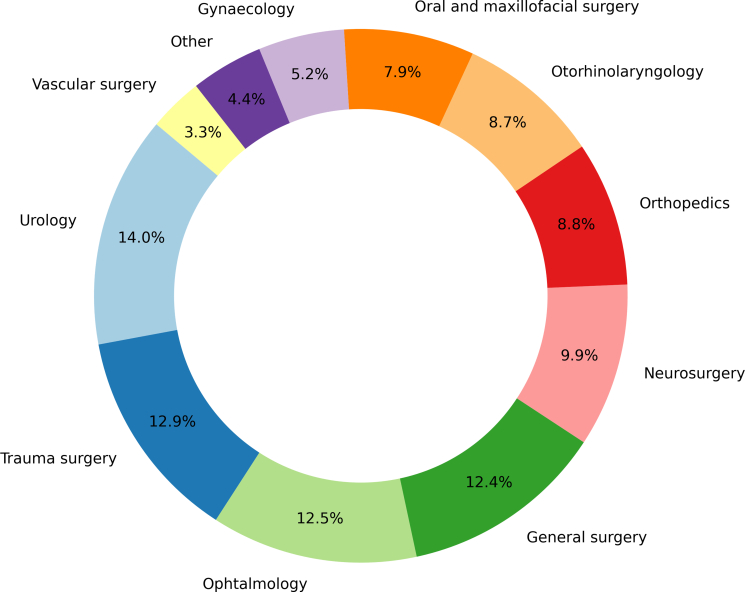
Surgery departments of the total cohort.

Urological surgeries were performed in 14.0% of the overall cohort, 12.9% underwent trauma surgery, and 12.5% underwent ophthalmological procedures (Supplementary Fig. S2). A total of 1986 patients (79.6%) received only NIBP monitoring, whereas 511 patients (20.4%) received IBP monitoring during surgery (Supplementary Table S2).

### Intraoperative hypotension

A total of 5360 hypotensive events were recorded. Of all patients, 56.2% experienced at least one IOH event, with 627 (56.1%) in the robust cohort, 665 (56.4%) in the prefrail cohort, and 105 (53.8%) in the frail cohort. The frequency during surgery was 2.06 hypotensive events for the robust, 2.17 for the prefrail, and 2.23 for the frail cohort. For anaesthesia induction, the frequency was 0.35 hypotensive events for the robust, 0.45 for the prefrail, and 0.43 for the frail cohort. Details of additional IOH endpoints are presented in Table 2. Values for additional hypotension cut-offs are presented in Supplementary Table S3.

**Table 2:** Outcome parameters for intraoperative hypotension. Data are presented as median (interquartile range). Categorical or ordinal variables are presented as frequencies or percentages.

Category	All (*n*=2495)	Robust (*n*=1116)	Prefrail (*n*=1184)	Frail (*n*=195)
Blood pressure measurements per surgery (*n*)	26 (17–40)	26 (17–40)	26 (17–40)	29 (18–42)
Blood pressure measurements per anaesthesia induction (*n*)	5 (3–7)	4 (3–7)	5 (3–8)	5 (3–8)
Number of hypotensive events per surgery (*n*)	5360	2321	2601	438
Number of hypotensive events per anaesthesia induction (*n*)	1012	390	537	85
Frequency of hypotensive events per surgery (*n*)	2.13	2.06	2.17	2.23
Frequency of hypotensive events per anaesthesia induction (*n*)	0.40	0.35	0.45	0.43
Time-weighted duration of hypotensive events per surgery (%)	3.3	3.4	3.2	3.2
Time-weighted area under MAP <65 mm Hg per surgery (mm Hg min^−1^)	0.1	0.1	0.1	0.2
Time-weighted duration of hypotensive events per anaesthesia induction (%)	3.9	3.5	4.3	3.8
Time-weighted area under MAP <65 mm Hg per anaesthesia induction (mm Hg min^−1^)	0.2	0.2	0.2	0.2

**Fig 2:**
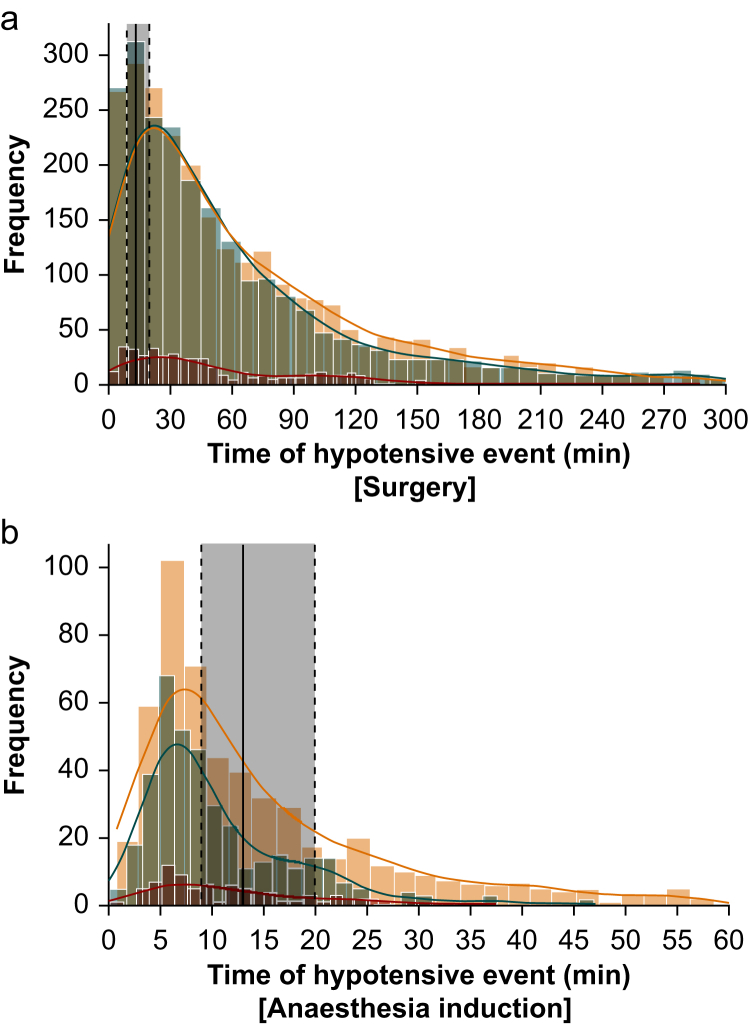
Rate of hypotensive events for the total surgery (a) and anaesthesia induction time (b) over time (min) in relation to frailty status. *Shaded brown* median and interquartile range of anaesthesia induction time of the overall cohort; *blue* robust; *yellow* prefrail; *red* frail; *coloured lines* Kernel Density Estimation.

Figure 2 illustrates the temporal occurrence of IOH during surgery (Fig. 2a) and anaesthesia induction (Fig. 2b), categorised by frailty status. A substantial portion of hypotensive events occurred immediately after the start of anaesthesia induction.

### Regression analysis

Logistic regression analysis revealed no significant difference in the likelihood of hypotensive events between groups during the entire surgery and anaesthesia induction (Table 3). In multivariate Poisson regression, a 9% increase in the rate of IOH for prefrail patients (IRR 1.09 [95% CI 1.03–1.16], *P*=0.002) and a 16% increase for frail patients (IRR 1.16 [1.04–1.29], *P*=0.007) for the entire surgical duration was demonstrated (Table 3). For anaesthesia induction, prefrail patients exhibited a 28% increase in the rate of IOH (IRR 1.28 [1.12–1.47], *P*<0.001), whereas the effect was not significant for frail patients (IRR 1.23 [0.96–1.56], *P*=0.10) (Table 3). Similar results were observed for MAP values <60 mm Hg but not for MAP values <55 mm Hg and <50 mm Hg (Supplementary Table S4). The results of the sensitivity analysis for only the first surgery per patient showed similar results of IOH (Supplementary Table S5). The sensitivity analysis, where coherent measurements counted as one IOH event, can be found in Supplementary Table S6. In a comparison between patients with NIBP *vs* IBP monitoring, the IBP group exhibited 46% (IRR 0.54 [0.50–0.59], *P*<0.001) fewer hypotensive events during surgery (Supplementary Table S7).

**Table 3:** Univariate and multivariate logistic and Poisson regression for surgery and anaesthesia induction time in relation to the probability of occurrence and rate of hypotensive events in (pre-)frail patients compared with the robust patient cohort. Factors besides frailty status of the multivariate analyses were age, ASA physical status, and BMI. IRR, incidence rate ratio; OR, odds ratio.

Surgery (probability of occurrence)	Univariate logistic regression OR (95% CI)	*P*-value	Multivariate logistic regression OR (95% CI)	*P*-value
Prefrailty	0.97 (0.83–1.15)	0.76	1.00 (0.85–1.19)	0.99
Frailty	0.90 (0.66–1.22)	0.50	0.94 (0.69–1.29)	0.71

Surgery (rate of hypotensive events)	Univariate poisson regression IRR (95% CI)	*P*-value	Multivariate Poisson regression IRR (95% CI)	*P*-value
Prefrailty	1.05 (0.99–1.11)	0.07	1.09 (1.03–1.16)	0.002
Frailty	1.08 (0.98–1.20)	0.13	1.16 (1.04–1.29)	0.007

Anaesthesia (probability of occurrence)	Univariate logistic regression OR (95% CI)	*P*-value	Multivariate logistic regression OR (95% CI)	*P*-value
Prefrailty	1.16 (0.96–1.40)	0.12	1.16 (0.96–1.42)	0.13
Frailty	1.10 (0.78–1.57)	0.58	1.10 (0.77–1.58)	0.59

Anaesthesia (rate of hypotensive events)	Univariate Poisson regression IRR (95% CI)	*P*-value	Multivariate Poisson regression IRR (95% CI)	*P*-value
Prefrailty	1.29 (1.14–1.48)	<0.001	1.28 (1.12–1.47)	<0.001
Frailty	1.25 (0.99–1.58)	0.06	1.23 (0.96–1.56)	0.10

For the time-weighted duration of IOH during surgery, multivariate Poisson regression showed an 11% decrease for prefrail patients (IRR 0.89 [95% CI 0.85–0.93], *P*<0.001). Frail patients did not show a significant difference for time-weighted duration of IOH during surgery (Table 4). For the time-weighted severity of IOH during surgery, no significant difference was observed between the two groups. During anaesthesia induction, prefrail (IRR 1.22 [1.16–1.27], *P*=0.001) and frail (IRR 1.15 [1.06–1.24], *P*<0.001) patients experienced a 22% and 15% longer time-weighted duration of IOH, respectively (Table 4). These showed no significant differences in time-weighted severity compared with robust cohort.

**Table 4:** Univariate and multivariate Poisson regression for surgery and anaesthesia induction time in relation to the duration, time-weighted duration, severity (area under MAP <65 mm Hg), and time-weighted severity of hypotensive events in (pre-)frail patients compared with the robust patient cohort. The time-weighted duration, or severity, was defined as the duration, or severity, divided by the respective surgery or anaesthesia induction time. Factors besides frailty status of the multivariate analyses were age, ASA physical status, and BMI. IRR, incidence rate ratio; OR, odds ratio.

	Univariate Poisson regression IRR (95% CI)	*P*-value	Multivariate Poisson regression IRR (95% CI)	*P*-value
**Surgery**				
Duration				
Prefrailty	0.98 (0.94–1.02)	0.26	1.04 (0.99–1.08)	0.09
Frailty	0.95 (0.88–1.02)	0.14	1.05 (0.97–1.13)	0.25
Time-weighted duration				
Prefrailty	0.87 (0.83–0.90)	<0.001	0.89 (0.85–0.93)	<0.001
Frailty	0.94 (0.87–1.02)	0.12	1.00 (0.92–1.08)	0.99
Severity				
Prefrailty	1.04 (1.02–1.06)	<0.001	1.14 (1.12–1.17)	<0.001
Frailty	1.21 (1.16–1.25)	<0.001	1.42 (1.37–1.47)	<0.001
Time-weighted severity				
Prefrailty	0.88 (0.71–1.09)	0.24	0.92 (0.73–1.15)	0.48
Frailty	1.19 (0.82–1.71)	0.36	1.30 (0.89–1.91)	0.17
**Anaesthesia induction**				
Duration				
Prefrailty	1.38 (1.25–1.53)	<0.001	1.34 (1.20–1.49)	<0.001
Frailty	1.29 (1.07–1.55)	0.007	1.22 (1.01–1.47)	0.04
Time-weighted duration				
Prefrailty	1.21 (1.16–1.26)	<0.001	1.22 (1.16–1.27)	<0.001
Frailty	1.14 (1.06–1.23)	0.001	1.15 (1.06–1.24)	0.001
Severity				
Prefrailty	1.25 (1.19–1.30)	<0.001	1.19 (1.13–1.24)	<0.001
Frailty	1.18 (1.08–1.28)	<0.001	1.07 (0.98–1.16)	0.12
Time-weighted severity				
Prefrailty	1.18 (0.99–1.41)	0.07	1.17 (0.97–1.41)	0.09
Frailty	1.13 (0.81–1.56)	0.48	1.11 (0.79–1.55)	0.56

The effect of the rate of IOH was attributed to the respective frailty status to over 90%, even after considering the number of blood pressure measurements (prefrail: 91.5% [87.0%–96.1%], *P*<0.001; frail: 92.3% [81.5%–100.0%], *P*<0.001) and the duration of surgery (prefrail: 99.0% [93.7%–100.0%], *P*<0.001; frail: 100.0% [88.6%–100.0%], *P*<0.001) (Supplementary Table S8). When considering the total propofol dose normalised to body weight, the effect was attributed to the respective frailty status to >70% (prefrail: 73.0% [0.0%–100.0%], *P*=0.32; frail: 70.2% [56.5%–84.0%], *P*<0.001). Similar results were observed for consideration of fluids, norepinephrine, and surgery departments.

## Discussion

In this retrospective cohort study, frailty status was not associated with a higher likelihood of IOH during surgery or induction of anaesthesia. However, in the adjusted analysis, prefrail and frail patients experienced more hypotensive events than robust patients during surgery. Although there were no differences in the severity of IOH if surgery or anaesthesia induction duration was taken into account, frailty status demonstrated a longer duration of IOH during induction of anaesthesia in the time-weighted analysis.

This is the first study to identify frailty as a risk factor for IOH. The impact of IOH on postoperative adverse outcomes was explored in a systematic review encompassing 42 studies,^5^ which showed that MAP <65 mm Hg was associated with increased mortality, acute kidney injury, stroke, and myocardial injury. These risks increased with duration and number of hypotensive events. A MAP <65 mm Hg for >20 min was linked to a high risk of end-organ injuries.^5^ Our data show that patients with frail status had a 16% higher frequency of hypotensive events during surgery compared with robust patients. Consequently, they were exposed to significantly more hypotensive events and were at higher risk of developing end-organ injuries. A potential contributing factor to IOH might be use of vasodilatory agents, such as propofol, during induction of anaesthesia. We speculate that robust patients exhibit prompt restoration to normotensive MAP and enhanced haemodynamic stability throughout the surgical procedure.

As a particularly vulnerable phase for IOH, induction of anaesthesia was identified in a retrospective cohort study of 42 825 patients, investigating the association of hypotension, defined as a MAP <65 mm Hg, during noncardiac surgery before and after skin incision.^9^ Our data show that 20% of all hypotensive events during surgery were attributed to induction of anaesthesia. In this context, (pre-)frail patients exhibited 22% and 15% longer hypotensive phases, respectively. This highlights the importance of individualised risk-adapted strategies for induction of anaesthesia, especially in relation to frailty status.

Jor and colleagues^26^ examines risk factors for IOH occurring immediately after induction of general anaesthesia. Their prospective multicentre study involving 661 patients demonstrated that age significantly correlated with IOH shortly after intubation and at 5 and 10 min after anaesthesia induction. Similarly, Reich and colleagues^27^ identified general risk factors for IOH in their retrospective study of 4096 patients undergoing general anaesthesia. An ASA physical status of 3–4 and induction of anaesthesia with propofol were recognised as contributing factors. The ASA physical status solely assesses severity of pre-existing conditions. In contrast, assessing frailty status provides the opportunity to capture more comprehensive patient-associated risk factors, such as diminished functional capacity in daily activities.^15^ Both studies pointing to age and ASA physical status as risk factors for IOH may be biased as they have not corrected for frailty or functional status.^26^^,^^27^ Our data, even after adjusting for age and ASA physical status in the multivariate analysis, indicate that frail patients experienced an increase in hypotensive events of 16% during the entire surgical procedure.

Frailty screening can thus contribute to classifying the vulnerable group of older patients and identifying those who have a significantly increased risk for a poor outcome. Birkelbach and colleagues^14^ showed that routine preoperative frailty assessment predicts postoperative complications in older patients across various surgical disciplines. Interestingly, prefrail patients exhibited a significantly shorter time-weighted duration of hypotensive events by 11% overall. However, when considering the induction alone, this cohort showed a marked increase in time-weighted duration of hypotensive events by 22%. Greater hypotension during induction might lead to haemodynamic stabilisation, thus reducing total duration of hypotension during surgery.

A comparison between patients with NIBP or IBP monitoring revealed that the IBP group experienced a 46% lower rate of IOH during surgery. Similar results were found by Wax and colleagues^28^ who reported that hypotensive patients with NIBP monitoring during surgery required vasopressors 11% less frequently than with IBP monitoring. This might suggest that vulnerable patient groups, such as (pre-)frail patients, could benefit from continuous invasive haemodynamic monitoring, as hypotensive phases can be detected more quickly and receive more adequate haemodynamic stabilisation.

A possible preventive approach would increase the physiological reserves of frail patients by prehabilitation,^29^^,^^30^ which addresses deficits before surgery by enhancing strength, flexibility, and endurance through preoperative physical therapy.^31^^,^^32^ However, the impact of prehabilitation on hypotensive events in (pre-)frail patients has not been investigated.

This study was retrospective, and is thus susceptible to unmeasured confounding, and was performed in a single centre, limiting generalisability. Nevertheless, we tried to address confounders in the multivariate or mediator analysis and sensitivity analyses. In terms of generalisability, the study was performed in a large university hospital, with a broad range of surgical disciplines included in the analysis. Another risk of bias is the substantial number of excluded patients, which was owing to the use of routinely collected data. Although the number of frail patients is limited, there are no substantial differences in the results between prefrail and frail patients, indicating that both groups are at a higher risk. Blood pressure data were transmitted to the electronic patient records at intervals as short as 2 min, introducing gaps between measurements that could not be considered in the statistical analysis.

## Conclusions

In this retrospective cohort study, frailty status was not associated with a higher likelihood of having an IOH event during surgery or anaesthesia induction or its severity, while there was an association with a higher frequency of hypotensive events during surgery. During induction of anaesthesia, frailty status was associated with longer duration, whereas only prefrailty, but not frailty, was associated with a higher frequency, which should be investigated in further prospective studies. Preoperative optimisation and modification of intraoperative management have the potential to reduce IOH. This could involve enhancing physiological reserves by prehabilitation, or adapting perioperative management accordingly, for example using IBP measurement to achieve better haemodynamic stabilisation.

## Authors’ contributions

Conceptualisation: ND, LH, CS, AP, JK, OH, BW, FB, RM, SJS

Methodology: ND, LH, CS, AP, JK, OH, BW, FB, RM, SJS

Validation: ND, LH, AB, NL, ND, MM, DvW, RM, SJS

Coding: ND, LH

Formal analysis: ND, LH, RM

Data curation: ND, LH, AB, NL, ND, MM, DvW, RM

Original draft: ND

Visualisation: ND

Review and editing: LH, CS, AP, JK, OH, BW, FB, AB, NL, ND, MM, DvW, RM, SJS

Supervision: CS, SJS

Project administration: SJS

Reviewed the final manuscript and consent to its publication in its current form: all authors

## Declaration of interest

The authors declare that they have no conflicts of interest.
