# Supplementary material for: Influence of frailty status on the incidence of intraoperative hypotensive events in elective surgery: Hypo-Frail, a single-centre retrospective cohort study
Source: Br J Anaesth. 2025 Jan 24;135(1):40–7. doi: 10.1016/j.bja.2024.10.050 (PMC12598938; doi:10.1016/j.bja.2024.10.050)
Supplement: Multimedia component 1 [file mmc1.pdf]

Supplementary data

Influence of frailty status on the incidence of intraoperative hypotensive events in elective surgery: a single-centre retrospective cohort study (Hypo-Frail)

Nils Daum[1], Laerson Hoff[1], Claudia Spies[1], Anne Pohrt[2], Annika Bald[1], Nadine Langer[1], Jörn Kiselev[3], Nils Drewniok[1], Maximilian Markus[1], Oliver Hunsicker[1], Rudolf Mörgeli[1], Björn Weiss[1], Dario von Wedel[4], Felix Balzer[4], Stefan J. Schaller[1,5*]

[1] Charité – Universitätsmedizin Berlin, Corporate Member of Freie Universität Berlin and Humboldt Universität zu Berlin, Department of Anaesthesiology and Intensive Care Medicine (CCM/CVK), Berlin, Germany

[2] Charité – Universitätsmedizin Berlin, Corporate Member of Freie Universität Berlin and Humboldt Universität zu Berlin, Institute for Biometry and Clinical Epidemiology, Berlin, Germany

[3] Fulda University of Applied Sciences, Department of Health Sciences, Fulda, Germany

[4] Charité – Universitätsmedizin Berlin, Corporate Member of Freie Universität Berlin and Humboldt Universität zu Berlin, Institute for Medical Informatics, Berlin, Germany

[5] Medical University of Vienna, Department of Anaesthesia, Intensive Care Medicine and Pain Medicine, Division of General Anaesthesia and Anaesthesiological Intensive Care Medicine

***Corresponding author:**

Prof. Dr. Stefan Schaller
Charité – Universitätsmedizin Berlin
Charitéplatz 1
10117 Berlin
Germany
stefan.schaller@charite.de

X(Twitter): @DaumNils; @DrStefan2

Table of contents

Supplementary Tables 3
Table S1 3
Table S2 4
Table S3 4
Table S4 5
Table S5 6
Table S6 7
Table S7 7
Table S8 8
Supplementary Figures 9
Figure S1 9
Figure S2 10
References 11

Supplementary Tables

Table S1

Table S1: Frailty criteria, adapted from Fried et al.[1] and Birkelbach et al.[2] Slight modifications were made to Fried's frailty assessment to adapt and improve data collection according to European standards, as summarized previously.[3] These modifications included estimating weight loss in kilograms instead of pounds and using a cutoff of ≥5 kg instead of ≥10 pounds (approximately 4.5 kg). Additionally, metabolic equivalent tasks (METs) were used instead of kilocalories per week (kCal/w). According to Fried, it is important to classify physical activity into low, moderate, and high levels, with a low level of activity in kCal/w being cited as a pathological criterion.[1]

Frailty Criteria	Description			
Shrinking: weight loss	Unintentional weight loss ≥5 kg within the previous year			
Weakness: reduced grip strength (dominant hand), by sex and BMI	*Male*		*Female*	
	BMI ≤24	≤29 kg	BMI ≤23	≤17 kg
	BMI 24.1 - 26	≤30 kg	BMI 23.1 - 26	≤17,3 kg
	BMI 26.1 - 28	≤30 kg	BMI 26.1 - 29	≤18 kg
	BMI >28	≤32 kg	BMI >29	≤21 kg
Exhaustion: answering C or D to the following question	How often in the past week did the following apply:<br>"I felt that everything I did was an effort."<br>"I could not get going."<br>a) Never or rarely<br>b) Sometimes<br>c) Often<br>d) Most of the time			
Gait Speed: slow walking speed (15 ft. = 4,57 m), dynamic start, by sex and height	*Male*		*Female*	
	Height ≤173 cm	≥7 s	Height ≤159 cm	≥7s
	Height >173 cm	≥6 s	Height > 159 cm	≥6s
Low activity	Metabolic Equivalent Tasks < 3			

Number of positive criteria: Frail: ≥3 criteria; Pre-frail: 1–2 criteria; *BMI* Body Mass Index

Table S2

Table S2. Comparison of non-invasively and invasively measured blood pressure values for overall cohort.

Category	NIBP (n = 1986)	NIPB & IBP (n = 511)
Surgeries (n)	2000	513
Blood pressure measurements per surgery (n)	29 [19 – 44]	17 [11 – 27]
Number of hypotensive events per surgery (n)	4713	644
Frequency of hypotensive events per surgery (n)	2.4	1.3

NIBP Non invasive blood pressure; *IBP* Invasive blood pressure

Table S3

Table S3. Number and frequency of different cut off values for intraoperative hypotension of the entire cohort, as well as robust, prefrail and frail patients.

Category	All (n = 2495)	Robust (n = 1116)	Prefrail (n = 1184)	Frail (n = 195)
Number of MAP <60 mmHg events per surgery (n)	1928	829	918	181
Number of MAP <55 mmHg events per surgery (n)	657	298	307	52
Number of MAP <50 mmHg events per surgery (n)	235	109	111	15
Frequency of MAP <60 mmHg events per surgery (n)	0.77	0.74	0.77	0.92
Frequency of MAP <55 mmHg events per surgery (n)	0.26	0.27	0.26	0.27
Frequency of MAP <50 mmHg events per surgery (n)	0.09	0.10	0.09	0.08

Categorical or ordinal variables are presented as frequencies.

Table S4

Table S4. Univariate and multivariate logistic and Poisson regression for surgery time in relation to the probability of occurrence (a, c ,e) and frequency of hypotensive events with different cut off values (b, d, f) in (pre-)frail patients compared to the robust patient cohort. Factors besides frailty status of the multivariate analyses were age, ASA status, and BMI.

a (MAP <60 mmHg)	Univariate Logistic Regression OR (95% CI)	p-value	Multivariate Logistic Regression OR (95% CI)	p-value
Pre-Frailty	1.04 (0.87 – 1.23)	0.69	1.05 (0.88 – 1.26)	0.58
Frailty	1.15 (0.84 – 1.58)	0.39	1.89 (0.85 – 1.65)	0.31

b (MAP <60 mmHg)	Univariate Poisson Regression IRR (95% CI)	p-value	Multivariate Poisson Regression IRR (95% CI)	p-value
Pre-Frailty	1.04 (0.95 – 1.15)	0.38	1.10 (1.01 – 1.22)	0.04
Frailty	1.25 (1.07 – 1.47)	0.006	1.38 (1.17 – 1.63)	<0.001

c (MAP <55 mmHg)	Univariate Logistic Regression OR (95% CI)		Multivariate Logistic Regression OR (95% CI)	
Pre-Frailty	1.01 (0.81 – 1.27)	0.88	1.04 (0.82 – 1.31)	0.74
Frailty	1.05 (0.70 – 1.59)	0.80	1.09 (0.71 – 1.67)	0.69

d (MAP <55 mmHg)	Univariate Poisson Regression IRR (95% CI)		Multivariate Poisson Regression IRR (95% CI)	
Pre-Frailty	0.97 (0.83 – 1.14)	0.71	1.04 (0.88 – 1.23)	0.63
Frailty	1.00 (0.75 – 1.34)	0.10	1.12 (0.83 – 1.52)	0.46

e (MAP <50 mmHg)	Univariate Logistic Regression OR (95%-CI)	p-value	Multivariate Logistic Regression OR (95%-CI)	p-value
Pre-Frailty	1.03 (0.75 – 1.42)	0.84	1.09 (0.78 – 1.52)	0.62
Frailty	1.05 (0.58 – 1.89)	0.87	1.14 (0.62 – 2.10)	0.67

f (MAP <50 mmHg)	Univariate Poisson Regression IRR (95% CI)	p-value	Multivariate Poisson Regression IRR (95% CI)	p-value
Pre-Frailty	0.96 (0.74 – 1.25)	0.76	1.04 (0.79 – 1.37)	0.77
Frailty	0.78 (0.46 – 1.35)	0.39	0.90 (0.52 – 1.57)	0.72

ASA-Status American Society of Anesthesiologists Physical Status System; *BMI* Body-Mass-Index; *IRR* Incidence Rate Ratio; *OR* Odds Ratio

Table S5

Table S5. Univariate and multivariate logistic and Poisson regression for surgery (a, b) and anaesthesia induction time (c, d) in relation to the probability of occurrence and rate of hypotensive events in (pre-)frail patients compared to the robust patient cohort for the first surgery per patient

a	Univariate Logistic Regression OR (95% CI)	p-value	Multivariate Logistic Regression OR (95% CI)	p-value
Pre-Frailty	0.98 (0.83 – 1.15)	0.76	1.00 (0.85 – 1.19)	0.98
Frailty	0.91 (0.67 – 1.23)	0.54	0.95 (0.69 – 1.30)	0.75
**b**	**Univariate Poisson Regression IRR (95% CI)**	**p-value**	**Multivariate Poisson Regression IRR (95% CI)**	**p-value**
Pre-Frailty	1.05 (0.99 – 1.11)	0.08	1.09 (1.03 – 1.16)	0.003
Frailty	1.09 (0.98 – 1.20)	0.12	1.16 (1.05 – 1.29)	0.006
**c**	**Univariate Logistic Regression OR (95% CI)**	**p-value**	**Multivariate Logistic Regression OR (95% CI)**	**p-value**
Pre-Frailty	1.16 (0.96 - 1.40)	0.13	1.16 (0.96 - 1.42)	0.13
Frailty	1.11 (0.78 - 1.57)	0.57	1.11 (0.78 - 1.60)	0.56
**d**	**Univariate Poisson Regression IRR (95% CI)**	**p-value**	**Multivariate Poisson Regression IRR (95% CI)**	**p-value**
Pre-Frailty	1.29 (1.13 – 1.47)	<0.001	1.28 (1.12 – 1.47)	<0.001
Frailty	1.26 (0.99 – 1.59)	0.06	1.24 (0.97 – 1.58)	0.08

IRR Incidence Rate Ratio; *OR* Odds Ratio

Table S6

Table S6. Univariate and multivariate logistic and Poisson regression for surgery (a, b) in relation to the probability of occurrence and rate of hypotensive events in (pre-)frail patients compared to the robust patient cohort, with coherent measurements counted as one event

a	Univariate Logistic Regression<br>OR (95% CI)	p-value	Multivariate Logistic Regression<br>OR (95% CI)	p-value
Pre-Frailty	1.02 (0.87 – 1.21)	0.78	1.05 (0.89 – 1.25)	0.56
Frailty	0.96 (0.71 – 1.31)	0.81	1.01 (0.74 – 1.39)	0.93

b	Univariate Poisson Regression<br>IRR (95% CI)	p-value	Multivariate Poisson Regression<br>IRR (95% CI)	p-value
Pre-Frailty	1.03 (0.96 – 1.11)	0.46	1.07 (0.98 – 1.16)	0.11
Frailty	0.96 (0.83 – 1.11)	0.60	1.02 (0.88 – 1.19)	0.78

IRR Incidence Rate Ratio; *OR* Odds Ratio

Table S7

Table S7. Univariate and multivariate Poisson regression in comparison of non-invasively to invasively measured blood pressures during surgery for the overall cohort.

	Univariate Poisson Regression<br>IRR (95% CI)	p-value	Multivariate Poisson Regression<br>IRR (95% CI)	p-value
IBP	0.53 (0.49 – 0.58)	<0.001	0.54 (0.50 – 0.59)	<0.001

IRR Incidence Rate Ratio; *IBP* Invasive blood pressure

Table S8

Table S8. Mediator analysis for direct effect of (pre-)frailty on the rate of hypotensive events

	Direct effect	p-value	Mediator effect	p-value
*Measurements**				
Pre-Frailty	91.5% (87.0% - 96.1%)	<0.001	8.5% (8.0% - 8.9%)	<0.001
Frailty	92.3% (81.5% - 100.0%)	<0.001	7.7% (5.8% - 9.7%)	<0.001
*Surgery Time***				
Pre-Frailty	99.0% (93.7% - 100.0%)	<0.001	1.4% (1.2% - 1.6%)	<0.001
Frailty	100.0% (88.6% - 100.0%)	<0.001	0.0% (0.0% - 0.4%)	<0.001
*Propofol****				
Pre-Frailty	73.0% (0.0% - 100.0%)	n.s.	28.0% (0.0% - 79.0%)	<0.001
Frailty	70.2% (56.5% - 84.0%)	<0.001	29.8% (19.2% - 42.7%)	<0.001
*Fluids*****				
Pre-Frailty	95.1% (90.4% - 99.8%)	<0.001	0.1% (0.1% - 0.1%)	<0.001
Frailty	100.0% (89.0% - 100.0%)	<0.001	0.0% (0.0% - 0.0%)	n.s.
*Noradrenaline******				
Pre-Frailty	100.0% (96.2% - 100.0%)	<0.001	0.0% (0.0% - 0.0%)	n.s.
Frailty	100.0% (100.0% - 100.0%)	<0.001	0.0% (0.0% - 0.0%)	n.s.
Surgery departments				
Pre-Frailty	100.0% (96.8% - 100.0%)	<0.001	0.0% (0.0% - 0.7%)	n.s.
Frailty	100.0% (100.0% - 100.0%)	<0.001	0.0% (0.0% - 0.0%)	<0.001

The overall effect was standardized to 100% and the direct and mediator effects were calculated proportionally. * Number of measured blood pressures during surgery time; ** Total surgery time; *** Amount of propofol (mg kg^{-1}) during surgery; **** Amount of fluids (ml kg^{-1} min^{-1}); ***** Amount of Noradrenaline (µg kg^{-1}); n.s. non-significant

Supplementary Figures

Figure S1

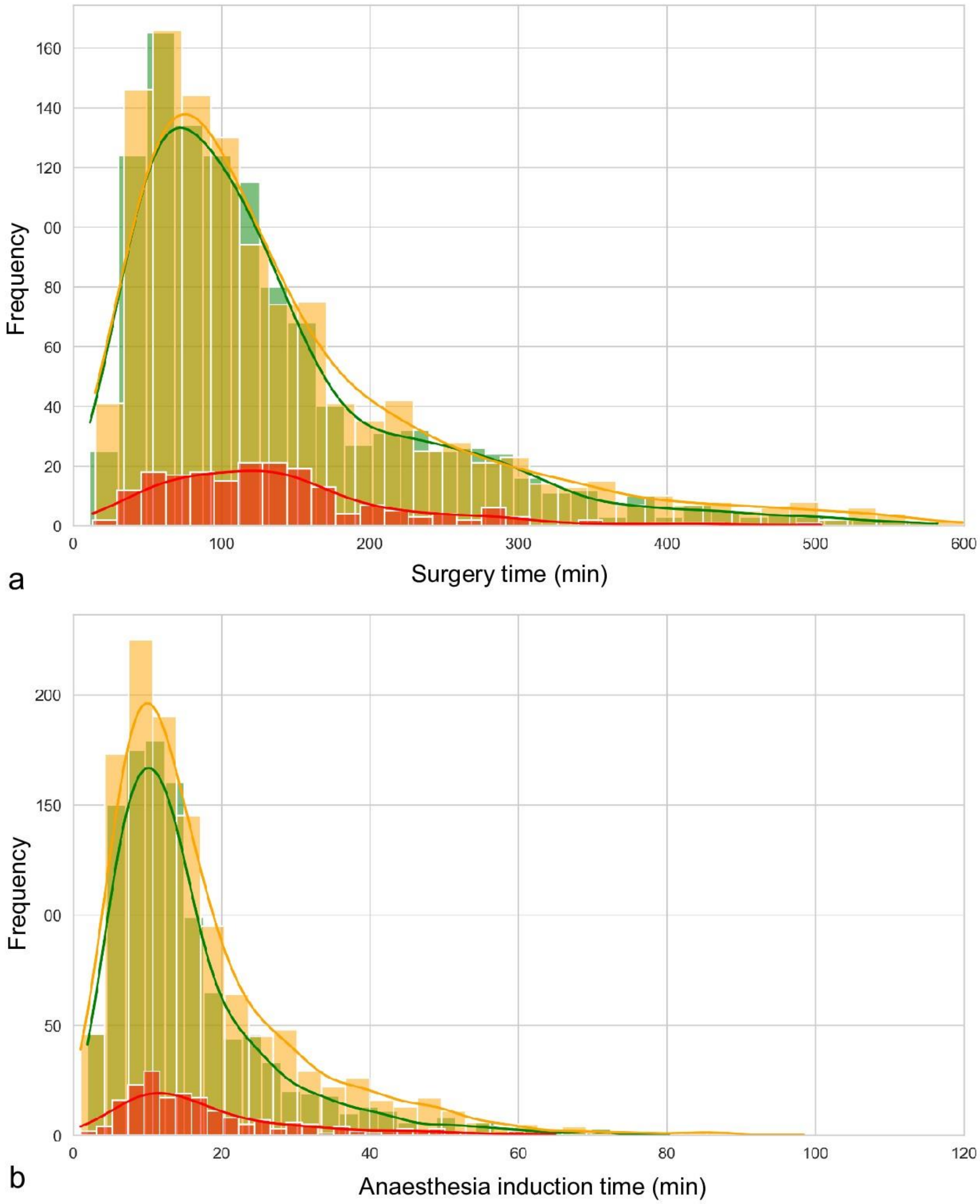

Figure S1. Total surgery (a) and anaesthesia induction time (b). *green* robust; *yellow* prefrail; *red* frail; *coloured lines* Kernel Density Estimation

Figure S2

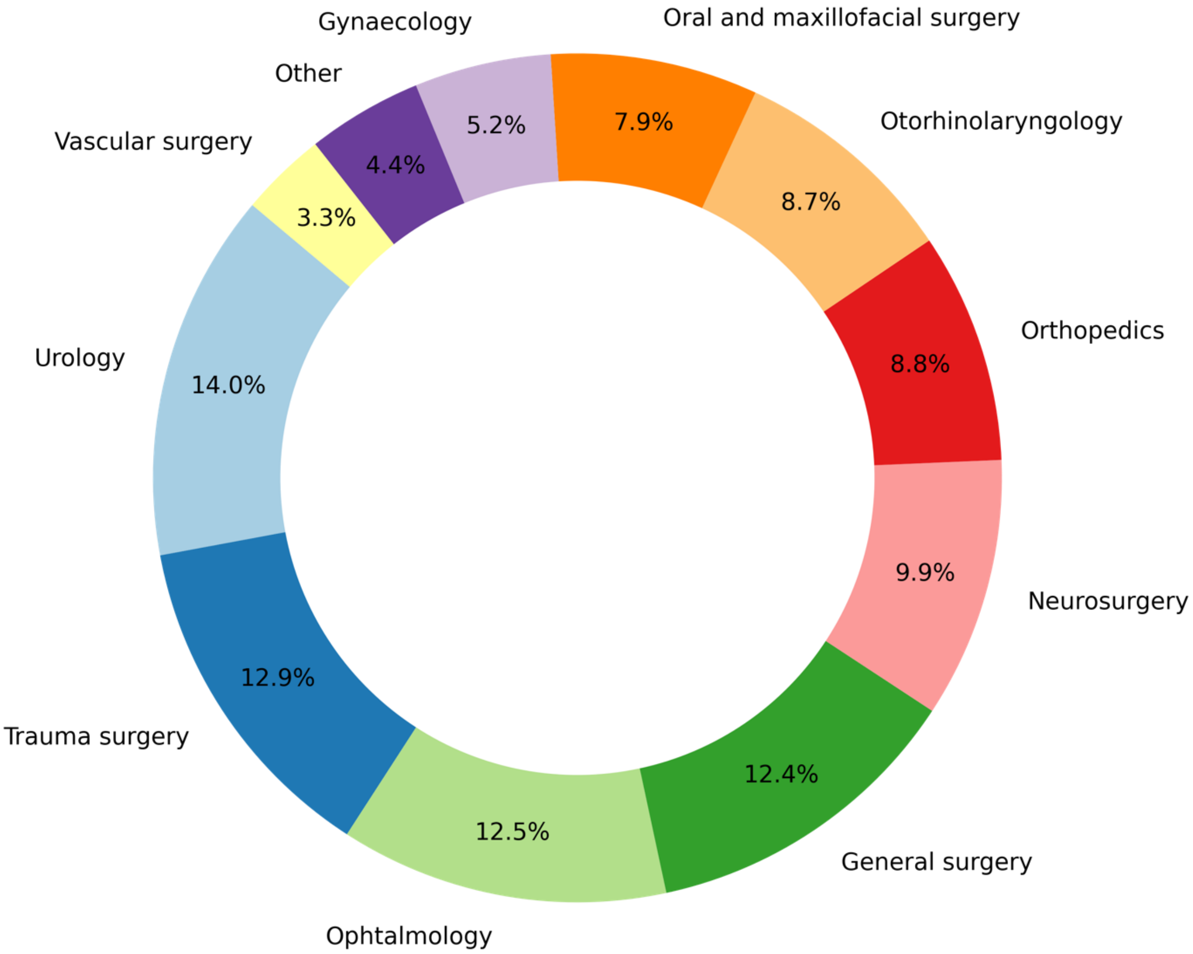

Figure S2. Surgery departments of the total cohort

References

1 Fried LP, Tangen CM, Walston J, et al. Frailty in older adults: evidence for a phenotype. *J Gerontol A Biol Sci Med Sci* 2001; **56**: M146-56

2 Birkelbach O, Mörgeli R, Spies C, et al. Routine frailty assessment predicts postoperative complications in elderly patients across surgical disciplines - a retrospective observational study. *BMC Anesthesiol* 2019; **19**: 204

3 Birkelbach O, Mörgeli R, Balzer F, et al. [Why and How Should I Assess Frailty? A Guide for the Preoperative Anesthesia Clinic]. *Anasthesiol Intensivmed Notfallmed Schmerzther* 2017; **52**: 765-76
